# Engineering a d-lactate dehydrogenase that can super-efficiently utilize NADPH and NADH as cofactors

**DOI:** 10.1038/srep24887

**Published:** 2016-04-25

**Authors:** Hengkai Meng, Pi Liu, Hongbing Sun, Zhen Cai, Jie Zhou, Jianping Lin, Yin Li

**Affiliations:** 1 Department of Cellular Biology, University of Science and Technology of China, Hefei, China; 2 CAS Key Laboratory of Microbial Physiological and Metabolic Engineering, Institute of Microbiology, Chinese Academy of Sciences, Beijing, China; 3 Tianjin Institute of Industrial Biotechnology, Chinese Academy of Sciences, Tianjin, China; 4 College of Pharmacy, Nankai University, Tianjin, China

## Abstract

Engineering the cofactor specificity of a natural enzyme often results in a significant decrease in its activity on original cofactor. Here we report that a NADH-dependent dehydrogenase (d-LDH) from *Lactobacillus delbrueckii* 11842 can be rationally engineered to efficiently use both NADH and NADPH as cofactors. Point mutations on three amino acids (D176S, I177R, F178T) predicted by computational analysis resulted in a modified enzyme designated as d-LDH*. The K_cat_/K_m_ of the purified d-LDH* on NADPH increased approximately 184-fold while the K_cat_/K_m_ on NADH also significantly increased, showing for the first time that a rationally engineered d-LDH could exhibit comparable activity on both NADPH and NADH. Further kinetic analysis revealed that the enhanced affinity with NADH or NADPH and the significant increased K_cat_ of d-LDH* resulted in the significant increase of d-LDH* activity on both NADPH and NADH. This study thus demonstrated that the cofactor specificity of dehydrogenase can be broadened by using targeted engineering approach, and the engineered enzyme can efficiently function in NADH-rich, or NADPH-rich, or NADH and NADPH-rich environment.

Dehydrogenases are important enzymes involved in the biosynthesis of chemicals. In nature, most microbial dehydrogenases prefer to use a single molecule, either NADH or NADPH, as its main cofactor. Sufficient supply of reducing equivalent, NADH or NADPH, and their efficient regeneration, are crucial for production of bulk chemicals via metabolic engineering^1^,^2^,^3^,^4^. However, except for a few examples, for instance glutamate dehydrogenase which is active on both NADH and NADPH^5^, most natural dehydrogenases prefer to use either NADH, or NADPH, as its main cofactor. Usually NADH is the major reducing equivalent in heterotrophic microorganism, such as *E. coli*, while NADPH is the major reducing equivalent in photoautotrophic microorganisms, such as cyanobacteria^4^,^6^. This means NADPH-dependent enzyme may not function well in *E. coli*. For example, introduction of the NADPH-dependent ketol-acid reductoisomerase into *E. coli* did not contribute to production of isobutanol production^1^. Similarly, NADH-dependent enzyme, for instance the NADH-dependent lactate dehydrogenase (LDH), may not work well in cyanobacteria^2^,^3^,^4^. Development of an enzyme that can efficiently use NADH or NADPH as cofactor would be useful, as the activity of such an enzyme will not be limited by the availability of NADH or NADPH.

Cofactor specificity can be altered by co-expression of a transhydrogenase, or both a transhydrogenase and a NAD kinase, to accelerate the interconversion between NADH and NADPH^1^,^2^,^4^. This strategy increased the production of isobutanol^1^ and lactate^2^,^4^. Nevertheless, manipulations of the expression levels of transhydrogenase and NAD kinase were required to meet the specific cofactor requirement^1^. Cofactor specificity can also be changed via site-directed mutagenesis. For instance, engineering the cofactor specificity of the NADH-dependent l-lactate dehydrogenase (l-LDH)^3^ and d-lactate dehydrogenase (d-LDH)^7^. However, the activity of the engineered LDHs on NADPH was much lower than that of the wild-type on NADH, and the activity of the engineered LDHs on NADH was also significantly decreased^3^,^7^. This suggests that the engineered LDHs are of little practical value, although the cofactor specificity was successfully altered.

Recently, biosynthesis of lactate from biomass or carbon dioxide becomes very attractive, with the hope of producing biodegradable polymer polylactide (PLA) to address the global energy and environment challenges^2^,^3^,^4^,^8^,^9^,^10^,^11^. l-lactate and d-lactate are two optical isomers of lactate, whereas, d-lactate is the essential moiety for the thermostability of PLA^9^,^10^. Biosynthesis of d-lactate is more difficult than that of l-lactate because the d-lactate dehydrogenase (d-LDH) is not widely present in nature^12^. Almost all natural d-LDH characterized to date are dependent on NADH^13^, which makes production of d-lactate in NADPH-rich microbes difficult.

The aim of this study was to investigate whether it is possible to broaden the cofactor specificity of the d-LDH without decreasing its activity on original cofactor. We used a rational engineering strategy based on enzyme structure comparison and analysis to engineer the NADH-dependent d-LDH from *Lactobacillus delbrueckii* 11842, which shows the highest d-LDH activity reported to date^7^,^10^,^14^. Using the rational engineering strategy, we obtained an engineered d-LDH which showed great activity on both NADH and NADPH.

## Results

### Identification of the target sites for engineering d-LDH

**Figure 1:**
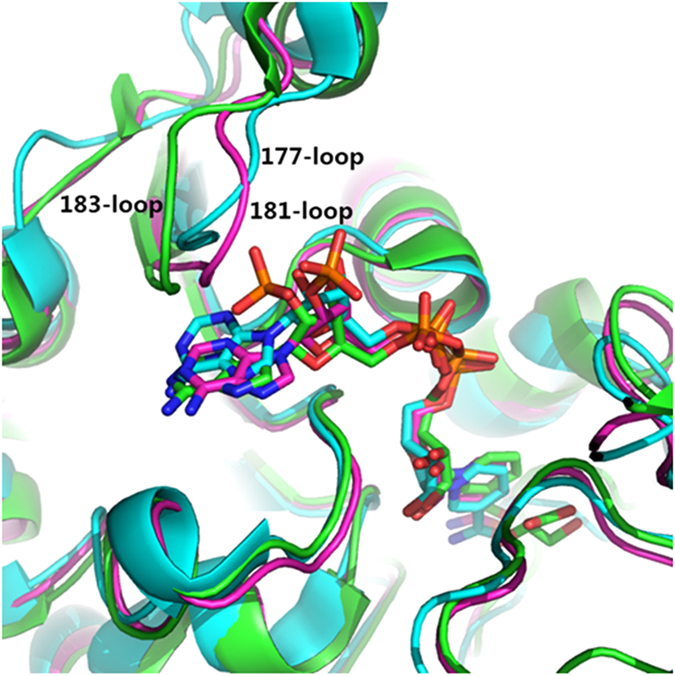
Alignment of three pdb, 1J49 (cyan), 2DBQ (magenta) and 2GCG (green). The only notable difference between these three binding pocket is at 177-loop of 1J49 (181-loop of 2DBQ, 183-loop of 2GCG). Abbreviations: pdb, protein data ban; 1J49, d-LDH which is NADH-dependent enzyme; 2DBQ and 2GCG, glyoxylate reductases which are NADPH-dependent enzymes.

It is known that the structure of the cofactor binding pocket determines the cofactor specificity^15^,^16^. Comparing the structure of the NADH binding pocket and the NADPH binding pocket may help to reveal the difference, thus providing clues for rational design to alter the cofactor specificity. d-LDH is an NADH-dependent enzyme and 1J49^15^ is the crystal structure of d-LDH. To find NADPH-dependent enzyme with structure similar to that of 1J49, we subjected the sequence of 1J49 (Fig. S1) to RCSB PDB and the results were sorted by similarity. Two types of NADPH bound crystal structures were selected: glyoxylate reductase 2DBQ^17^ (EC 1.1.1.26, E-Value: 8.12976E-17, Identities: 73/259 (28%), Positives: 121/259 (47%), Gaps: 25/259 (10%)) and 2GCG^18^ (EC 1.1.1.79, E-value: 2.23185E-15, Identities: 73/294 (25%), Positives: 126/294 (43%), Gaps: 23/294 (8%)). To reveal the difference on the selectivity of the binding pockets, the three crystal structure of 1J49, 2DBQ and 2GCG were aligned and analyzed. The RMSD between 1J49 and 2DBQ is 1.958, the RMSD between 1J49 and 2GCG is 1.819. Because of the structure similarity of NADH and NADPH, the binding pockets structure and residues type of 1J49, 2DBQ and 2GCG are also highly similar if not identical (Fig. 1 and Fig. S2). The only notable structure difference between NADH and NADPH binding pockets is the 177-loop in 1J49 (181-loop in 2DBQ and 183-loop in 2GCG). In this selectivity control loop, 2DBQ has the same residues number with 1J49 (YSRTR:YDIFR), whereas the 2GCG has one more residue in this loop (YTGRQP: YDIFR). Furthermore, the 181-loop of 2DBQ has a higher sequence similarity with that of the 177-loop of 1J49: the residues at the beginning and the end are the same, while the three residues located in the middle are different. As one of the key objectives of engineering d-LDH is to minimize the impact on NADH-dependent activity, we thus selected the 181-loop of 2DBQ as a template to engineer the d-LDH (1J49) to minimize the structure instability.

**Figure 2:**
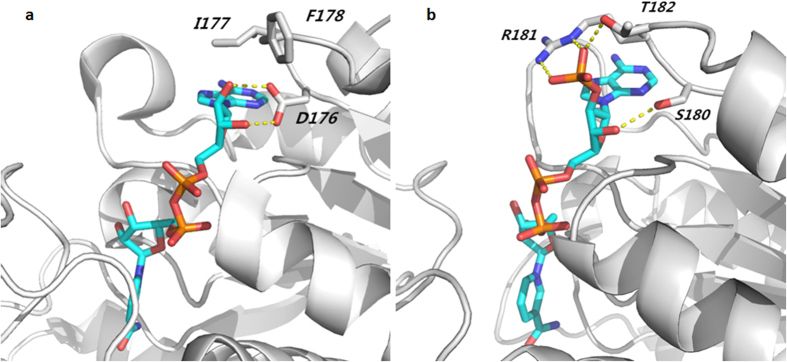
Interaction analysis of the two notable difference loops with its prefer cofactor. (**a**) Interaction analysis of 177-loop of 1J49 with NAD^+^. D176 has two hydrogen-bond with ribose of NAD^+^ and residue I177 has hydrophobic interaction with purine ring. (**b**) Interaction analysis of 181-loop of 2DBQ with NADP^+^. R181 and T182 have three hydrogen bond with phosphate group of NADP^+^. Residue S181 has one hydrogen bond with ribose of NADP^+^.

In 177-loop of 1J49, the residue D176 has two hydrogen-bonds with the ribose of NAD^+^ (Fig. 2a), while residue I177 has hydrophobic interaction with the purine ring. In 181-loop of 2DBQ, the residues R181 and T182 have three hydrogen bonds with the phosphate group of NADP^+^ (Fig. 2b), while the residue S180 has one hydrogen bond with the ribose of NADP^+^. All these three middle residues in 2DBQ have hydrogen bond interaction with NADP^+^. Therefore, introducing mutations into all these three residues are expected to change the cofactor specificity of 1J49 (EC 1.1.1.28), and the YDIFR loop of d-LDH will be replaced with the YSRTR loop, which prefers to bind NADPH.

### Engineering cofactor binding pocket enables d-LDH to efficiently use NADPH

**Figure 3:**
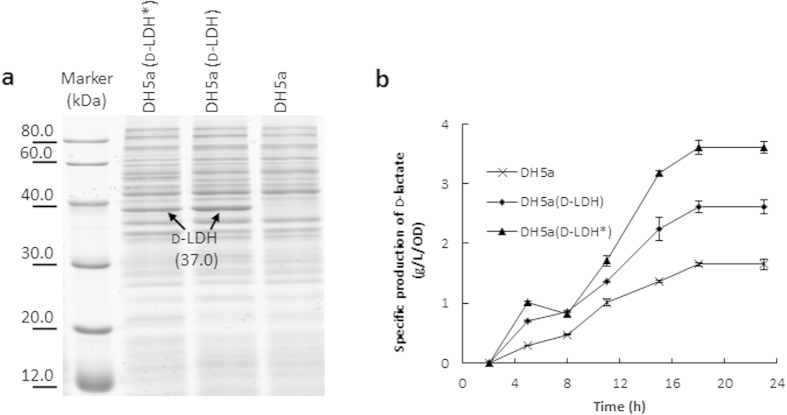
Crude enzymatic activity analysis of d-LDH and d-LDH*. (**a**) Detection of *d-LDH* and d-LDH* gene expression in corresponding strain via 12% SDS-PAGE analysis. Lane 1, Protein Markers; Lane 2, DH5a (d-LDH*); Lane 3, DH5a (d-LDH); Lane 4, DH5a. (**b**) Time course of d-lactate production.

To replace the YDIFR loop of 1J49 with the NADPH binding loop YSRTR, point mutations on three amino acids (D176S, I177R, F178T) were introduced into d-LDH, resulting in a mutant designated as d-LDH*. To investigate the effect of the loop replacement, the specific activity of crude d-LDH* using NADPH as cofactor was determined. As the enzyme has not yet been purified, the expression levels of d-LDH* and d-LDH in *E. coli* DH5α were investigated prior to the enzymatic activity assay. SDS-PAGE shows that similar expression levels of d-LDH* and d-LDH were achieved (Fig. 3a). As d-LDH is a NADH-dependent enzyme, the specific activity of wild-type d-LDH using NADPH as cofactor was very low (Table S1). The specific activity of d-LDH*, in which YDIFR was replaced with YSRTR, was significantly higher than d-LDH using NADPH as cofactor (Table S1), demonstrating that this loop replacement strategy is working. Compared to wild-type d-LDH, the specific activity of d-LDH* using NADPH as cofactor increased approximately 57-fold (Table S1) through this rational engineering approach.

### Engineered d-LDH^*^ also increases its activity on NADH

It is very common that enzyme engineering approaches altering the cofactor specificity usually result in a sharp decrease of the enzyme activity on original cofactor^7^. To investigate whether the natural enzymatic activity of d-LDH* on NADH was affected by replacing the loop YDIFR with YSRTR, the specific activities of the d-LDH* and wild-type d-LDH were assayed using NADH as cofactor. To our surprise, the specific activity of crude d-LDH* using NADH as cofactor was not decreased, but much higher than that of the wild-type d-LDH using NADH as cofactor (Table S1). The specific activity of crude d-LDH* using NADH as cofactor increased approximately 4-fold (Table S1) through this rational engineering approach. Moreover, the specific activity of crude d-LDH* using NADPH as cofactor (37.84 ± 3.33 μmol/min/mg crude cell extraction) was comparable with the specific activity of d-LDH* using NADH as cofactor (34.47 ± 3.81) μmol/min/mg crude cell extraction), indicating such a rational design successfully altered the cofactor specificity of d-LDH, and significantly altered its activities on both NADH and NADPH.

### Engineered d-LDH^*^ shows increased K_cat_/K_m_ values on both NADH and NADPH

To better understand the effect of this rational engineering approach on enzyme properties, d-LDH and d-LDH* with his_6_-tag at their C terminus were constructed, expressed in BL21(DE3), and purified by using Ni^2+^ affinity chromatography (Fig. S3). The purified d-LDH and d-LDH*were subjected to further characterization.

The specified activities of the purified his-tagged d-LDH and d-LDH* were shown in Table 1. The specific activity of purified d-LDH* using NADH as cofactor increased by 28% as compared to that of the purified wild-type d-LDH, demonstrating that such a rational engineering approach did not decrease the original enzyme activity indeed. The specific activity of purified d-LDH* using NADPH as cofactor increased approximately 62-fold compared to that of the purified wild-type d-LDH. Specific activity assay using crude cell extract and purified d-LDH* using NADPH as cofactor showed highly similar degree of increment, demonstrating the activity of d-LDH* on NADPH was greatly improved.

To further address why the engineered enzyme d-LDH* can efficiently use both NADH and NADPH as cofactors, the kinetic parameters of d-LDH* and its wild-type d-LDH were determined and comparably analyzed. As shown in Table 1, the K_cat_ value of d-LDH* for NADH increased slightly and K_m_ value decreased, leading to that the K_cat_/K_m_ value of d-LDH* for NADH increased by 52% compared with that of the d-LDH. Most strikingly, K_cat_/K_m_ value of d-LDH* for NADPH increased by 184-fold, due to the 9.6-fold increased K_cat_ and the 16.7-fold decreased K_m_, as compared with that of d-LDH. The purified d-LDH* exhibited a high and similar K_cat_ when using NADH or NADPH, suggesting the d-LDH* can catalyze dehydrogenation reaction in a similar rate no matter NADH or NADPH is used as cofactor. Moreover, the K_m_ of d-LDH* on NADH decreased by 38.6%, while the K_m_ of d-LDH* on NADPH was 16.7-fold lower than that of the d-LDH. This suggests the d-LDH* has acquired a significantly improved affinity on NADPH.

### Increased production of d-lactate in *E. coli* using d-LDH*

To further confirm the improvement of enzymatic efficiency of the engineered d-LDH* *in vivo*, the d-lactate production in *E. coli* DH5α cells expressing d-LDH or d-LDH* were investigated. Profiles of d-lactate production (Fig. 3b) showed that the production of d-lactate per OD cells reached the highest at 18 hour. The highest titer of the d-lactate produced by *E. coli* expressing the d-LDH* was 37% higher than that of expressing the original d-LDH, indicating that the *in vivo* activity of d-LDH* increased.

## Discussion

Creating enzymes with novel properties by protein engineering is an important approach for metabolic engineering, with the aim to increase the titers and productivities of target products, or to produce non-natural compounds^19^. In a previous work, NADH-dependent phenylalanine dehydrogenase was successfully developed by engineering an NADPH-dependent glutamate dehydrogenase to achieve partial biosynthesis of a nonproteinogenic amino acid^20^. In another work, d-lactate dehydrogenase activity was evolved from glycerol dehydrogenase by protein engineering and production of d-lactate from lignocellulose was achieved^21^.

Methods for protein engineering include random and targeted mutagenesis^19^. Efforts have been made to alter cofactor specificity of LDH from NADH to NADPH by using site-directed mutagenesis approach^3^,^7^,^22^. However, there are little successful examples of targeted mutagenesis for improving the properties of LDH. Target mutagenesis of the NADH-dependent d-LDH from *L. delbrueckii* 11842 on cofactor binding site D175A resulted in a moderate increase in catalytic activity on NADPH but a 7.5-fold decrease in catalytic activity on NADH^22^. In addition, targeted mutagenesis of NADH-dependent l-LDH from *B. subtilis* on cofactor binding site V38R resulted in a 50% decrease in catalytic activity on NADH and the activity on NADPH was only 1/6 of the wild-type activity on NADH^3^. Further analysis showed it was the improvement of the expression level of the l-LDH V38R mutant, rather than the improved specific activity, resulted in the increased lactate production in cyanobacteria^3^. In a word, although the engineered LDHs can use NADPH as cofactor, the activity was far too low to have practical significance.

Usually random mutagenesis and targeted mutagenesis are combined to improve an enzyme^20^. To date, the only successful example for engineering the NADH-dependent l-LDH from *B. subtilis* was achieved by using saturation mutagenesis. A point mutation on the cofactor binding site V39R resulted in a significant improvement of catalytic activity on NADH and a 100-fold increase of activity on NADPH^23^. However, the saturation mutagenesis based on five created mutation library, is more time-consuming and laborious than the rational engineering strategy described in our study.

The highlight of this work is that the rationally engineered d-LDH* can use both NADH and NADPH as cofactor, efficiently. Comparative analysis of the crystal structure of the NADH-dependent d-LDH and two other NADPH-dependent enzymes identified the only notable different loop of cofactor binding pockets. Subsequently, three amino acid residues were mutated based on the analysis of the interaction of cofactor with its binding loop. Benefited from this rational design, simple modification on three amino acid residues resulted in a 184-fold increased K_cat_/K_m_ on NADPH and a 52% increased K_cat_/K_m_ on NADH, as determined using the purified enzyme. d-LDH from *L. delbrueckii* 11842 is already the most active d-LDH reported to date in terms of its activity on NADH. The further increased activity on NADH, together with acquisition of the comparable activity on NADPH, have made the d-LDH* an attractive enzyme candidate for improving d-lactate production. Although the K_cat_/K_m_ value of d-LDH on NADPH could be increased by directed mutagenesis, they were usually at the cost of decreased K_cat_/K_m_ of d-LDH on NADH^7^,^22^ (Table 2). Here we showed an exception that the cofactor specificity of d-LDH can be shifted from NADH to NADPH without sacrificing its activity on original cofactor by directed mutagenesis.

**Figure 4:**
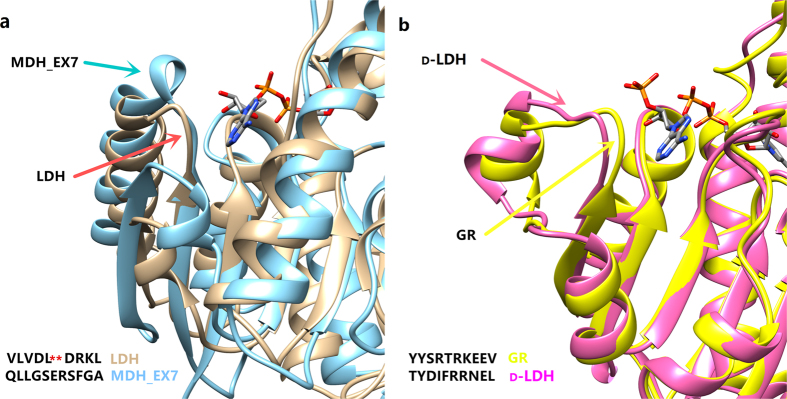
Alignment of crystal structure and sequence of cofactor binding loops. (**a**) MDH EX7(1WZI) and LDH(2E37). (**b**) d-LDH (1J49) and GR(2DBQ).

The selection of the template enzyme for rational design is crucial for the success of the targeted mutagenesis, while minimizing the difference on the structure of enzymes is the principle for minimizing the negative impact on original enzyme activity. In a previous study^24^, a NADPH binding loop region containing seven amino acid residues from a NADPH-dependent malate dehydrogenase was introduced into the cofactor binding site of the LDH from *Thermus thermophilus.* Although the cofactor specificity was shifted to NADPH, the activity on NADH was dramatically decreased. It seems likely that the authors^24^ did not consider the difference in crystal structures of the two proteins when deciding to replace the sequence “DLDRKLA” of LDH with sequence “GSERSFQ” of MDH_EX7. As shown in Fig. 4a, MDH_EX7 has a circle of helix and a loop binding to NADPH, whereas LDH only has a loop binding to NADH. Replacement of the sequence “DLDRKLA” with “GSERSFQ” might generate side-effects as the binding affinity of the substitute might be weakened by improper steric configuration. In such a case, we argue that replacing a shorter sequence “DLDR” of LDH with “GSERSF”, rather than replacing “DLDRKLA”, might generate a better result. The reason is that replacing a shorter sequence “DLDR” of LDH with “GSERSF” might build a similar 3D structure like MDH_EX7.

In this study, we selected the glyoxylate reductase (2DBQ) from *Pyrococcus horikoshii* OT3 for comparison with the d-LDH (1J49) from *L. delbrueckii* 11842, as the length of cofactor binding loops of these two enzymes are the same (Fig. 4b). The only notable difference between cofactor binding pockets of these two enzymes is the NADH binding loop YDIFR of d-LDH (1J49) and the NADPH binding loop YSRTR from 2DBQ. Such a minor modification may account for why the activity of d-LDH* on NADPH is comparable with that of wild-type d-LDH on NADH, which is superior compared with previous studies^3^,^22^,^24^. The structure of 1J49 and 2DBQ is almost identical except for the difference of the two loops, thus the impact of the targeted mutagenesis on protein structure was minimized. Besides the YDIFR loop, 1J49 also contains other NADH binding sites in its cofactor binding pocket^15^. The residue S180 in the substitute loop YSRTR may interact with the ribose group of NADH, which might explain the significantly increased activity of d-LDH* on NADH upon engineering.

Taken together, a novel d-LDH*, with much higher activity on NADPH and improved activity on NADH, was obtained based on our discovery of the only notable different loop between NADH and NADPH binding pockets. Such a superior d-LDH* will encourage researchers to use NADPH-rich cells, such as cyanobacteria, as hosts for d-lactate production. In addition, it provides an example to show how rational design can be used to engineer enzymes for altering its cofactor specificity without affecting its original activity.

## Materials and Methods

### Chemicals and reagents

All chemicals and solvents were purchased form Sigma-Aldrich unless otherwise specified. PrimeSTAR HS DNA Polymerase, pMD-18t-simple vector were purchased from Takara, while restriction enzyme and T4 DNA ligase were purchased from New England BioLabs.

### Crystal structure alignment and analysis

Crystal structures of 1J49^15^ (d-LDH), 2DBQ^17^ (a glyoxylate reductase from *Pyrococcus horikoshii* OT3) and 2GCG^18^ (a glyoxylate reductase from *Homo sapiens*) were retrieved from the RCSB protein data bank (www.pdb.org), respectively. Alignment of the structure of 1J49 , 2DBQ, and 2GCG were performed using UCSF chimera 1.10.1 software^25^ and the graph of protein structure was drawn using pymol1.5 software (The PyMOL Molecular Graphics System, Version 1.5 Schrödinger, LLC.).

### Strains and growth conditions

*E. coli* strain DH5α was used for construction of vectors. *E. coli* strains were cultured in LB medium at 37 °C in a shaking incubator at 200 rpm or on solidified LB plates containing 1.5% (w/v) agar. M9 medium supplemented with 30 g/L glucose as the sole carbon source and 100 mM MOPS as pH buffer was used for d-lactate production. For the measurements of d-lactate production, *E. coli* strains were first cultured in LB medium until exponential phase. Cells were then harvested by centrifugation at 5000 rpm for 6 min. The pellets were washed twice with M9 medium and re-suspended in M9 medium. These cultures were inoculated into 100 ml anaerobic bottles containing 30 ml M9 medium with an initial OD_600_ of 0.2, and cultivated at 37 °C. At selected time point, bacterial growth was measured by detecting the optical density at the wavelength of 600 nm and d-lactate production was determined by High Performance Liquid Chromatography (HPLC) described below.

### Computer-aided site-directed mutagenesis of d-LDH

The plasmids used and constructed in this study were listed in Table S2. The cloning vector pMD-18t-simple was served as a backbone for all plasmids constructed. pMD-Dldh^26^ containing P_cpc_-Dldh-T_rbcL_ DNA fragment, which consisted of the promoter P_cpc_, structural gene d-*ldh* and terminator T_rbcL_ was used as template. Site-directed mutagenesis was performed to introduce three continuous amino acids mutations to the d-*ldh*. Nucleotides AGTCGGACC were designed to replace GATATTTTT in the d-*ldh* to produced D176S (ASP176 → SER), I177R (ILE177 → ARG) and F178T (PHE178 → THR) described below. The mutated P_cpc_-Dldh*-T_rbcL_ DNA fragment was obtained by Overlap Extension PCR^27^. During the first round of PCR, forward primer P_cpc_-F (5′-ccgctcgagacctgtagagaagagtccct-3′) and reverse primer ldh*-R (substitution sites underlined, 5′-CTTCTCTAATTCGGGATTCCGGGTCCGACTATAGGCAATAACCTTCGCG-3′) were used to amplify the first intermediate fragment, and forward primer ldh*-F (substitution sites underlined, 5′-CGCGAAGGTTATTGCCTATAGTCGGACCCGGAATCCCGAATTAGAGAAG-3′) and reverse primer T_rbcl_-R (5′-CCGCTCGAGGCTGTCGAAGTTGAACATCA-3′) were used to amplify the second intermediate fragment, both with pMD-Dldh as the PCR template, these two intermediate fragments were mixed for the second round of PCR using flanking primers P_cpc_-F and T_rbcl_-R to create P_cpc560_-Dldh*-T_rbcL_. The P_cpc_-Dldh*-T_rbcL_ DNA fragment was excised using Xho1, followed by insertion into the corresponding site of pSM2^28^.

### Expression of protein and enzyme assays

Three milliliter *E. coli* DH5α cells with or without plasmids grown to OD_600_ of 2.5 were harvested by centrifugation at 14,000 rpm for 3 min and resuspended in 1 ml TE buffer (10 mM Tris-HCl, 1 mM EDTA pH = 8.0). Cells were broken using an ultrasonic cell disruptor on ice for 5 min and then removed the cell debris by centrifugation at 14,000 rpm for 3 min. Part of the supernatant was used to detect the protein concentration using Bradford method, and the rest was used to analyze the protein expression pattern on 12% SDS-PAGE.

Crude enzymatic activity assays for d-LDH and d-LDH* were performed as described in literature^26^ to determine the cofactor specificity, by monitoring the decrease in the absorbance of NADH or NADPH at 340 nm. The reaction mixture (200 μL) contained 150 mM sodium phosphate (pH 6.5), 300 μM NADH or NADPH, 2.5 mM MgCl_2_, and 3 μg crude extract protein. The reaction was initiated by addition of 30 mM sodium pyruvate, all reactions were performed in 96-well plate reader at 30 °C.

To further determine the kinetic parameters of d-LDH and d-LDH*, the two enzymes expressed in *E. coli* were purified using Immobilized Metal Affinity Chromatography (IMAC)^29^.

To determine the kinetic parameters of d-LDH and d-LDH*, the activity of d-LDH and d-LDH* under different concentrations of NADH or NADPH were determined. The reaction mixture (200 μL) contained 100 mM PBS buffer (pH 6.5), 2.5 mM MgCl_2_, 20 mM sodium pyruvate, and different concentrations of NADH or NADPH. One unit of enzyme activity was defined as the amount of enzyme required to convert 1 μmol NAD(P)H into NAD(P)^+^ per minute. Michaelis-Menten equation was used to calculate the kinetic parameters.

### Assay for lactic acid

The lactic acid concentrations in batch culture samples harvested at selected time points were analyzed by HPLC as described previously^30^. Briefly, cells were removed by centrifugation at 14,000 rpm for 2 min and then the supernatant (700 μL) was filtered and then analyzed by HPLC (Agilent 1260 Infinity Series), equipped with an Aminex HPX-87H ion exchange column (7.8 × 300 mm; Bio-Rad Laboratories, Inc., Hercules, CA, USA) and a refractive index (RI) detector (Agilent). 5 mM aqueous sulfuric acid was used as mobile phage at a flow rate of 0.50 ml/min at 15 °C.

## Additional Information

**How to cite this article**: Meng, H. *et al*. Engineering a d-lactate dehydrogenase that can super-efficiently utilize NADPH and NADH as cofactors. *Sci. Rep.* **6**, 24887; doi: 10.1038/srep24887 (2016).

## Supplementary Material

Supplementary Information

## Figures and Tables

**Table 1:** The kinetic parameters of the purified his-tagged LDH and LDH*.

Enzymes	Specific activity		Kinetic parameters						
	NADH (μmol/min/mg)	NADPH (μmol/min/mg)	NADH				NADPH		
			K_cat_ (s^−1^)	K_m_ (mM)	K_cat_/K_m_ (M^−1^s^−1^)	K_cat_ (s^−1^)	K_m_ (mM)	K_cat_/K_m_ (M^−1^s^−1^)	
d-LDH	1240 ± 38	49 ± 3	7801	1.40	5.57 × 10^6^	821	4.55	1.82 × 10^5^	
d-LDH*	1587 ± 52	3101 ± 47	8540	1.01	8.46 × 10^6^	8643	0.257	3.36 × 10^7^	

d-LDH, wild-type d-lactate dehydrogenase; d-LDH*, engineered d-lactate dehydrogenase. Data were relative to three independent measurements (±SD).

**Table 2:** Comparison of kinetic parameters for different wild-type LDH and corresponding mutants.

Enzyme	Mutated site	NADH		NADPH		References
		K_cat_ (s^−1^)	K_cat_/K_m_ (M^−1 ^s^−1^)	K_cat_ (s^−1^)	K_cat_/K_m_ (M^−1 ^s^−1^)	
LdhD	WT	561	2.4 × 10^5^	ND	ND	7
LdhDn^ARSdR^	D176A, I177R F178S, N180R	129	8.5 × 10^3^	163	4.4 × 10^4^	
L. bulgaricus d-LDH	WT	875	1.1 × 10^7^	425	2.6 × 10^5^	22
L. bulgaricus* d-LDH	D175A	575	1.15 × 10^6^	725	1.2 × 10^6^	
BsLDH	WT	77.5	1.5 × 10^6^	1.3	9.4 × 10^3^	23
BsLDH*	V39R	291.6	4.9 × 10^6^	140.4	2.3 × 10^6^	
Tt27LDH	WT	39.9	5.24 × 10^6^	5.93	4.33 × 10^4^	24
Tt27LDH-EX7	D^32^LDRKLA^38^	11.2	3.24 × 10^4^	127	1.07 × 10^5^	
	G^32^SERSFQ^38^					
d-LDH	WT	7801	5.57 × 10^6^	821	1.82 × 10^5^	This research
d-LDH*	D176S, I177R F178T	8540	8.46 × 10^6^	8643	3.36 × 10^7^	

ND: not determined.
