# Supplementary material for: Engineering a d-lactate dehydrogenase that can super-efficiently utilize NADPH and NADH as cofactors
Source: Sci Rep. 2016 Apr 25;6:24887. doi: 10.1038/srep24887 (PMC4842997; doi:10.1038/srep24887)
Supplement: Supplementary Information [file srep24887-s1.docx]

**Supplementary Information**

**Engineering a** **d-lactate dehydrogenase that can super-efficiently utilize NADPH and NADH as cofactors**

Hengkai Meng^†1, 2^, Pi Liu^†3^, Hongbing Sun^2,3^, Zhen Cai^2^, Jie Zhou^*2^, Jianping Lin^*3,4^, Yin Li^*2^

Table of Contents

1. **Table S1**. Specific activities of d-LDH and d-LDH* in crude cell extract.
2. **Table S2.** Strains and plasmids used in this study.
3. **Figure S1.** Amino acid sequence of d-LDH.
4. **Figure** **S2.** 3D-structure alignment of 1J49, 2DBQ (MSE edit to MET) and 2GCG.
5. **Figure S3.** 12% SDS-PAGE analysis of purified d-LDH and d-LDH*.

**Table S1 Specific activities of** **d-LDH and d-LDH* in crude cell extract.**

| Strains | Enzyme | Crude enzyme activity | |
| --- | --- | --- | --- |
|  |  | NADPH | NADH |
| DH5a (d-LDH) | d-LDH | 0.67±0.05 | 8.16±0.20 |
| DH5a (d-LDH***) | d-LDH* | 37.84±3.33 | 34.47±3.81 |

Specific activities were given in μmol/min/mg crude cell extract. d-LDH, wild-type d-lactate dehydrogenase; d-LDH*, engineered d-lactate dehydrogenase. Data were relative to three independent measurements (± SD).

**Table S2** Strains and plasmids used in this study.

| **Strains and plasmids** | **Description** | **Reference** |
| --- | --- | --- |
| **Strains**  *E.coli* DH5α  DH5α (d-LDH)  DH5α (d-LDH*)  **Plasmids**  pMD18-T-simple  pMD-DLDH  pMD-DLDH* | Commercial transformation host for cloning  *E.coli* DH5α containing pMD-DLDH  *E.coli* DH5α containing pMD-DLDH*  Amp^r^, *E. coli* cloning vector  pMD18-T derivate, Amp^r^ Km^r^, *DLDH* expression cassette  pMD18-T derivate, Amp^r^ Km^r^, *DLDH** expression cassette | TransGene  This study  This study  TakaRa  Previous study[^10^](#_ENREF_10)  This study |

*Abbreviations*: Amp^r^, ampicillin resistance; Km^r^, kanamycin resistance.


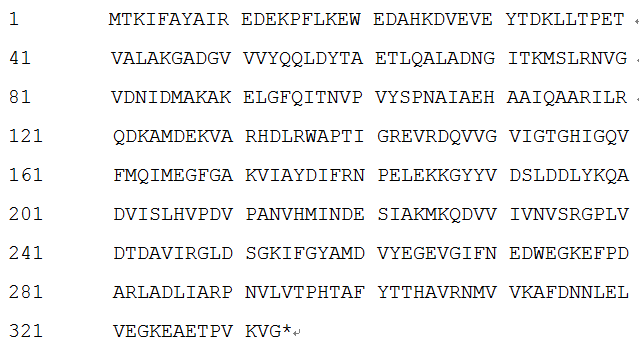


**Figure S1.** Amino acid sequence of d-LDH.


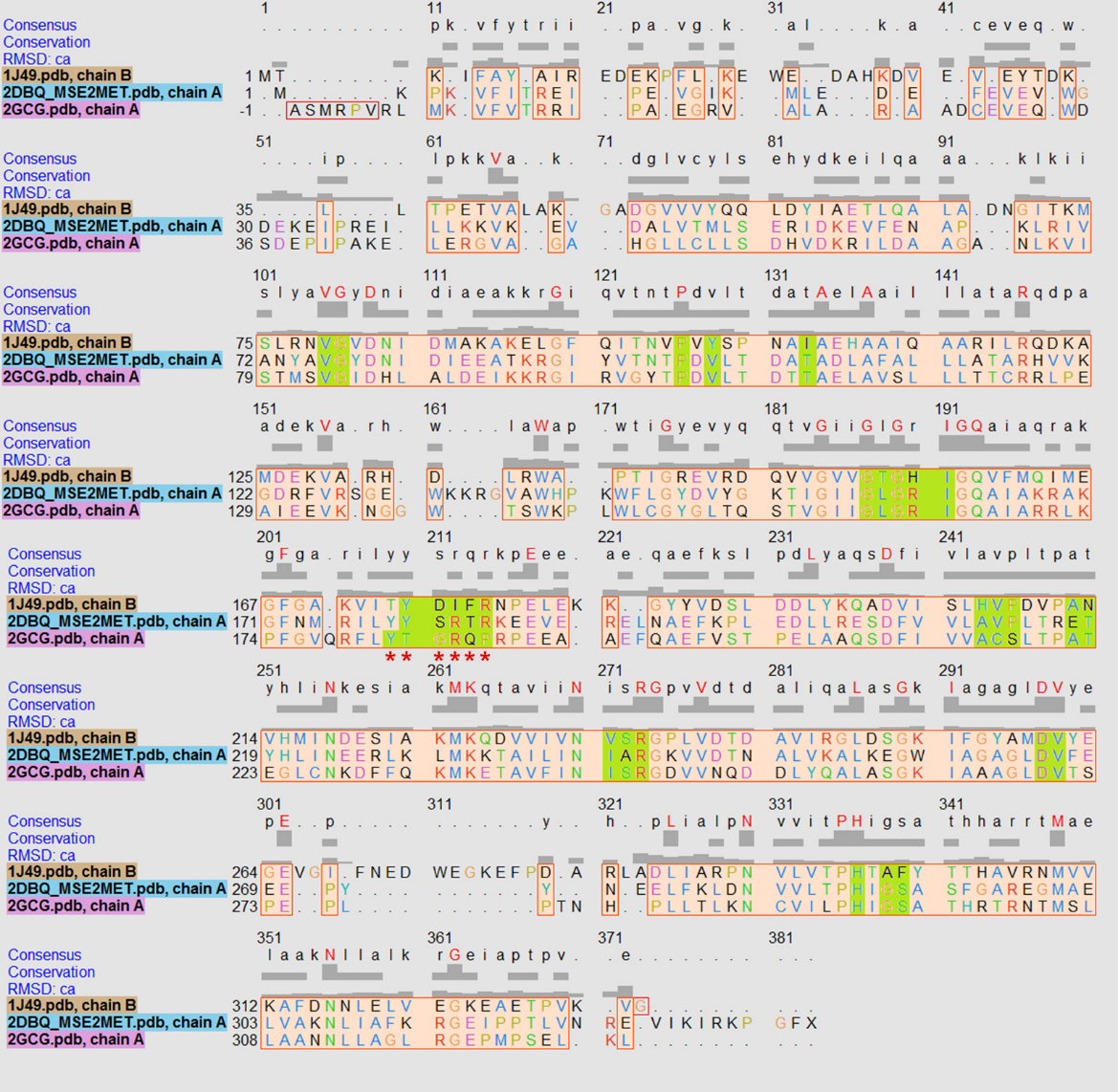


**Figure S2.** 3D-structure alignment of 1J49, 2DBQ (MSE edit to MET) and 2GCG. Residues adjacent to substitutes are color green. Conservation degree and RMSD of ca. are calculated and plotted. The 177-loop is pointed out by red “*”.


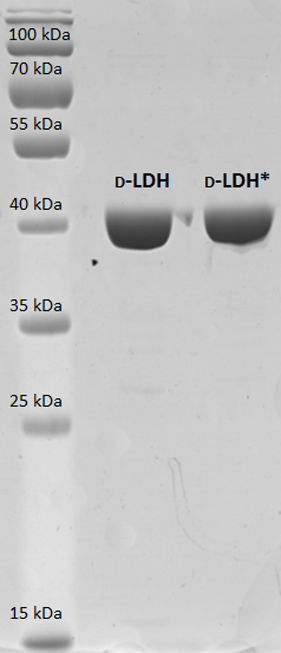


**Figure S3.** 12% SDS-PAGE analysis of purified d-LDH and d-LDH*.
